# Transcatheter arterial chemoembolization alone versus combined with microwave ablation for recurrent small hepatocellular carcinoma after resection: a retrospective comparative study

**DOI:** 10.1186/s12876-022-02387-7

**Published:** 2022-06-29

**Authors:** Jie Ji, Wei Yang, Hai-Bin Shi, Sheng Liu, Wei-Zhong Zhou

**Affiliations:** Department of Interventional Radiology, The First Affiliated Hospital of Nanjing Medical University, 300 Guangzhou Road, Gulou District, Nanjing, 210029 China

**Keywords:** Recurrence, Small hepatocellular carcinoma, Microwave ablation, Transcatheter arterial chemoembolization

## Abstract

**Purpose:**

To compare the efficacy and safety of transcatheter arterial chemoembolization combined with microwave ablation (TACE–MWA) versus TACE alone for the treatment of recurrent small hepatocellular carcinoma (sHCC) after resection.

**Materials and methods:**

From June 2015 to January 2020, a total of 45 patients with recurrent sHCC (size ≤ 3 cm) treated by TACE–MWA or TACE were included in this study. The radiological response at 1-, 3-, 6-month after initial treatment [modified Response Evaluation Criteria in Solid Tumors (mRECIST)], progression-free survival (PFS), overall survival (OS), and complications were evaluated.

**Results:**

The TACE–MWA group showed better 1-, 3-, 6-month tumor response rates than TACE group. The corresponding 1-, 3-, and 5-year PFS rates were 76.5%, 70.6%, and 70.6% for the TACE–MWA group, and 56.1%, 15.0%, and 15.0% for the TACE group (*P* = 0.003). The 1-, 3-, and 5-year OS rates were 100.0%, 82.1%, and 61.5% for the TACE–MWA group, and 89.0%, 58.1%, and 50.8% for the TACE group (*P* = 0.389), respectively. Moreover, no major complications related to treatment were observed in either of the groups. Compared with the TACE group, the TACE–MWA group had a significantly lower number of re-TACE sessions (*P* = 0.003).

**Conclusions:**

Although TACE alone provides equivalent effectiveness for recurrent sHCC in terms of OS rates, TACE–MWA had better 1-, 3-, 6-month tumor response rates and may prolong tumor PFS time.

## Introduction

Hepatic resection has been established as a curative treatment for hepatocellular carcinoma (HCC) [1–4]. However, the long-term prognosis of hepatic resection has been disappointing because of the high recurrence rates in the remnant liver [5–8]. Previous studies have reported that the cumulative 5-year recurrence rates after hepatic resection are as high as 78–96% [4, 9, 10]. Due to the close postoperative follow-up, as well as improved imaging technology, more recurrent small HCC (defined as ≤ 3 cm) are diagnosed at an early stage [11]. Therefore, the management of optimal treatment for recurrent sHCC is urgently required.

Repeat hepatectomy is an effective treatment for sHCC recurrence; however, it is limited by the patient’s general condition, remnant liver volume, tumor distribution, liver function, and tumor’s invasiveness. Mini-invasive therapies, such as radiofrequency ablation (RFA), microwave ablation (MWA) and TACE, have emerged as alternative treatments [5, 7, 9, 11]. The combination of TACE and ablation may have several theoretical advantages over either monotherapy [12–14]. First, the decreased blood flow induced by TACE reduces heat loss, thereby increasing the size of the ablation ablative zone. In addition, the lipiodol of TACE deposited in the tumor can be a mark for the ablation needle insertion and facilitates evaluation of the ablative margin [15]. TACE combined with RFA for sHCC has been shown to be superior to TACE alone [13]. Compared with RFA, MWA has less heat-sink effect and could achieve a larger coagulation volume in a shorter procedural time [16]. However, given limited literature, it remains unknown that whether TACE–MWA is effective in the treatment of recurrent sHCC.

The purpose of this study was to compare TACE–MWA with TACE alone for recurrent sHCC. We compared tumor response at 1 month, and progression-free survival (PFS), overall survival (OS), and complications after treatment with TACE–MWA or TACE alone.

## Materials and methods

### Patient selection

**Fig. 1:**
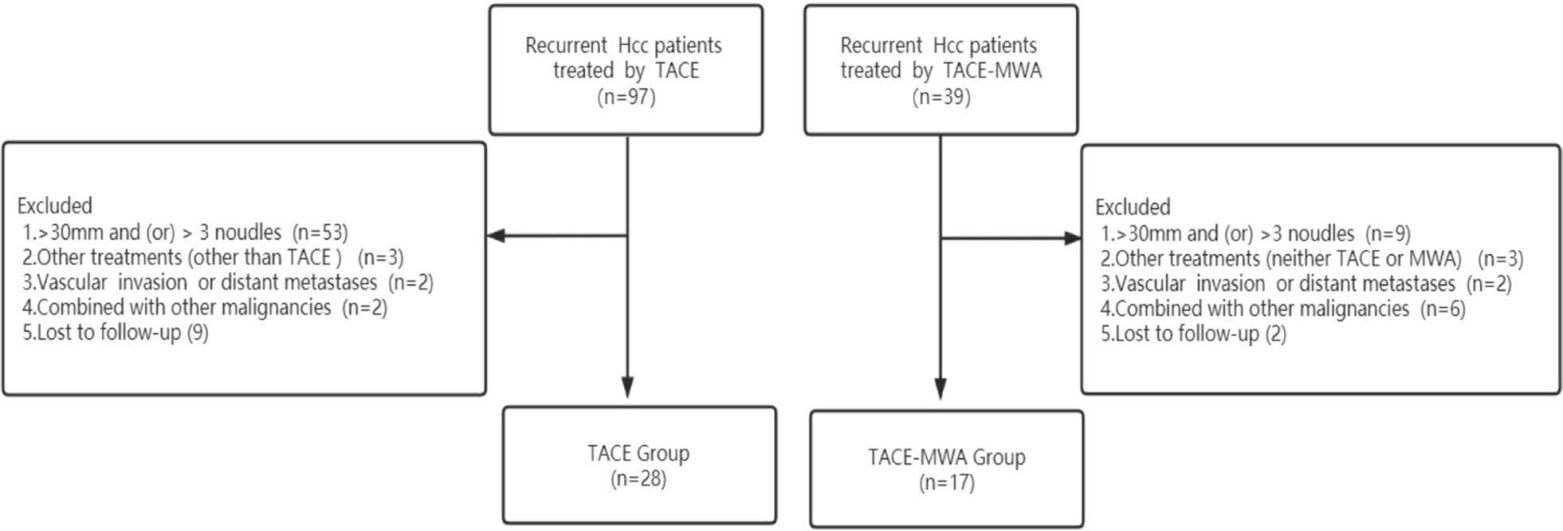
Flow chart of the study population

The study complied with the Declaration of Helsinki on human research and its later amendments and obtained approval of our institutional review board. Due to the retrospective study design, written informed consent was waived (the Ethics Committee of the First Affiliated Hospital with Nanjing Medical University). A total of 45 patients with recurrent sHCC treated with conventional TACE or TACE -MWA, were enrolled in our center from June 2015 to January 2020 (Fig. 1). The diagnosis of HCC was confirmed by pathology or imaging in accordance with the European Association for the Study of the Liver criteria guidelines. The inclusion criteria were as follows: (a) first intrahepatic recurrent HCC after initial resection, (b) recurrent three or fewer tumors under 3 cm prior to receiving TACE or TACE–MWA, (c) Child–Pugh A or B, (d) absence of extrahepatic metastasis or macrovascular invasion, and (e) Eastern Cooperative Oncology Group performance score ≤ 2. The exclusion criteria included: (a) additional treatments other than TACE or MWA, (b) secondary malignancies, (c) follow-up period less than 1 year or incomplete medical record.

### TACE

A 5F catheter was introduced through the femoral artery, to assess liver vascular anatomy and to confirm the patency of the portal vein by visceral angiography. Then, a selected micro-catheter (Terumo, Tokyo, Japan) was then super-selectively inserted into the hepatic lobe or hepatic segmental artery branch. A 1:1 mixed suspension of iodized oil (1–10 mL; Andre´ Guerbet Laboratories,) and epirubicin (20–40 mg; Pharmorubicin; Pfizer, Wuxi, China) were infused into the artery through the catheter, depending on the size and number of the tumor. Finally, the embolization was performed using a gelatin sponge until the blood supply of the tumor significantly decreased.

### TACE–MWA

After TACE procedures, MWA was applied sequentially within 2 weeks. MWA was performed using a microwave ablation system (ECO Corporation, Nanjing, China) at 2450 ± 50 MHz with continuous adjustable power output of 0–100 W. A plain scan of the tumor location was obtained by CT, to determine the optimal puncture trajectory. Under general anesthesia, the electrode probe was inserted to reach the opposite edge of the tumor through its center. The correct puncture position was ensured after an additional scan. Then, the microwave power and duration adjusted based on the tumor’s location and size, and the coagulation range was required to be 5–10 mm beyond the edge of the tumor. After the ablation, an addition CT scan was performed to verify the coagulation zone.

### Follow-up protocol

One month after the TACE–MWA or TACE procedure, each patient was followed by serum tumor makers, as well as enhanced liver magnetic resonance imaging (MRI) or CT. If no viable tumor was indicated, the follow-up intervals were extended to 2–3 months thereafter. If residual viable lesions were detected in the scan, repeated TACE was performed in the TACE group. Depending on tumor shape, size, or number, repeated TACE or MWA was performed in the TACE–MWA group. Based on mRECIST, we assessed PFS, which was defined as the interval from the first TACE treatment to tumor progression, death, or last follow-up. OS was defined as the interval from the first TACE treatment to death or last follow-up.

### Statistical analysis

The baseline characteristics of the patients were expressed as mean ± standard deviation (SD), median and interquartile range (IQR) or percentage. Comparisons between the two groups were done using the Student’s t test or Mann–Whitney U test for continuous data and the Chi-squared test or Fisher exact test were used for categorical variables. The PFS and OS were calculated by the Kaplan–Meier method and the groups were compared by log-rank test. All of the statistical tests were two-sided, and a difference was considered significant when P was lower than 0.05. The data were analyzed using SPSS (version 22.0; IBM Corp, Armonk, NY, USA) and R version 3.3.2 (R Foundation for Statistical Computing, Vienna, Austria).

## Results

### Patients’ characteristics

Clinical and histopathological data are summarized (Table 1). In this study, 15 patients (33.3%) had died and 30 patients (66.7%) were still alive. The median follow-up time for the cohort was 44.1 months. The single largest tumor size ranged from 0.8 to 3.0 cm. With respect to the number of tumors, 29 patients (64.4%) had 1 tumor, 12 patients (26.6%) had 2 tumors, and 4 patients (9.0%) had 3 tumors. In the TACE–MWA group, the largest single tumor size ranged from 1.0 to 3.0 cm. after the initial combined therapies, the mean re-TACE sessions was 1.4 (0 re-TACE: n = 8; 1: n = 4; 2: n = 3; 6: n = 1; 9: n = 1); and the mean re-MWA sessions was 0.2 (0 re-MWA: n = 13; 1: n = 2; 2: n = 1). In the TACE group, the single largest tumor size ranged from 0.8 to 3.0 cm. After the initial TACE procedure, the mean re-TACE sessions was 2.8 (0 re-TACE: n = 5; 1: n = 5; 2: n = 5; 3: n = 5; 4: n = 2; 6: n = 2; 7: n = 4).

**Table 1:** Baseline characteristics of patients

Variables	TACE (n = 28)	TACE – MWA (n = 17)	*P* value
Sex, n (%)			
Male	24	15	1.000
Female	4	2	
Age (years)	58.25 ± 10.15	60.29 ± 9.90	0.512
Initial HCC resection data			
Size of resected HCC, median (IQR), cm	3.5 (2.1–5.0)	3.0 (1.7–5.5)	0.277
No. of resected HCCs, median (IQR), n	1.0 (1.0–1.0)	1.0 (1.0–1.5)	0.353
Edmonson grade, n (%)			
I	12	8	0.572
II–III	15	7	
III	1	2	
BCLC stage, n (%)			
A	25	16	1.000
B	3	1	
Recurrence stage data			
Time to recurrence, median (IQR), days	319 (31–1478)	136(47–1872)	0.008
Largest tumor size, median (IQR), cm	1.6 (1.3–2.6)	1.4 (1.1–2.0)	0.452
Tumor number, median (IQR), n	1.0 (1.0–2.0)	1.0 (1.0–2.0)	0.289
HBV, n (%)			
Yes	28	17	NA
No	0	0	
AFP, n (%)			
≥ 400 ng/mL	5	2	0.693
< 400 ng/mL	23	15	
PLT, median (IQR), 10^9^/L	113.0 (74.7–154.5)	103.0 (84.0–159.0)	0.842
ALT, median (IQR), IU/L	25.1 (19.8–31.3)	20.5 (15.5–25.5)	0.055
AST, median (IQR), IU/L	28.5 (21.3–40.9)	22.9 (21.0–28.2)	0.075
ALB, median (IQR), g/L	39.0 (37.9–42.9)	40.0 (36.5–44.6)	0.870
TBIL, median (IQR), μmol/L	15.3 (11.9–18.8)	14.7 (12.7–18.3)	0.935
PT, median (IQR), s	12.6 (12.1–13.2)	12.5 (12.0–13.1)	0.439
INR, median (IQR)	1.1 (1.0–1.2)	1.1 (1.0–1.1)	0.439
Child–Pugh, n (%)			
A	26	17	0.519
B	2	0	

*IQR* interquartile range, *HBV* hepatitis B virus, *AFP* α-fetoprotein, *PLT* platelet, *PT* prothrombin time, *INR* international normalized ratio, *ALT* alanineaminotransferase, *AST* aspartate aminotransferase, *ALB* albumin, *TBIL* totbilirubin, *NA* not application, *TACE* transarterial chemoembolization, *MWA* microwave ablation, *BCLC* barcelona clinic liver cancer

### Radiological treatment response

Radiological response was assessed based on mRECIST criteria at 1-, 3-, 6-month after treatment [Table 2]. The 1-, 3-, and 6-month objective response rate (ORR) rates for the TACE–MWA and TACE alone group were 100% vs 71.4%, 88.2% vs 60.7%, and 82.3% vs 50%, respectively. The 1-, 3-, and 6-month disease control rate (DCR) rates for the TACE–MWA and TACE alone group were 100% versus 85.7%, 100% versus 71.4%, and 88.2% versus 67.9%, respectively. Evidently, the TACE–MWA group showed higher proportions of patients with ORR and DCR than the TACE group at any time point.

**Table 2:** Tumor response at 1, 3, 6 month between the two groups

Main outcome	One-month			Three-month			Six-month		
	TACE (n = 28)	TACE–MWA (n = 17)	*P* value*	TACE (n = 28)	TACE–MWA (n = 17)	*P* value*	TACE (n = 28)	TACE–MWA (n = 17)	*P* value*
Tumor response			0.044			0.020			0.011
CR	12 (42.8%)	14 (82.4%)		12 (42.8%)	14 (82.4%)		7 (25%)	13 (76.5%)	
PR	8 (28.6%)	3 (17.6%)		5 (17.9%)	1 (5.9%)		7 (25%)	1 (5.9%)	
SD	4 (14.3%)	0		3 (10.7%)	2 (11.7%)		5 (17.9%)	1 (5.9%)	
PD	4 (14.3%)	0		8 (28.6%)	0		9 (32.1%)	2 (11.7%)	
ORR	20 (71.4%)	17 (100%)		17 (60.7%)	15 (88.2%)		14 (50%)	14 (82.3%)	
DCR	24 (85.7%)	17 (100%)		20 (71.4%)	17 (100%)		19 (67.9%)	15 (88.2%)	

*TACE* transarterial chemoembolization, *MWA* microwave ablation, *CR* complete response, *PR* partial response, *SD* stable disease, *PD* progressive disease; objective response rate (ORR) = CR + PR; disease control rate (DCR) = CR + PR + SD

*Fisher exact test was used

### PFS

**Fig. 2:**
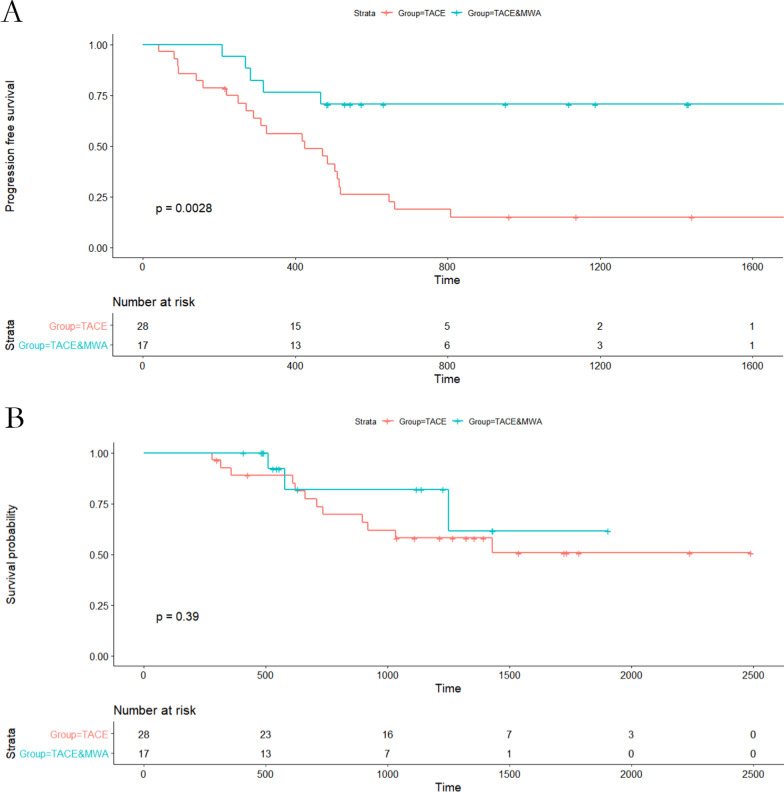
Progression-free survival (PFS) and overall survival (OS) curves with risk tables for patients with recurrent small HCC after hepatectomy who underwent TACE–MWA or TACE alone. **A** PFS curves. **B** OS curves

In the entire cohort, the median PFS was 16.8 months. There were 28 patients (62.2%) within the entire cohort experiencing tumor progression. In the TACE alone group, the median PFS was 14.1 months, with 23 patients (82.1%) having progression. In the TACE + MWA group, 5 patients (29.4%) had signs of progression at the time of analysis. The median PFS was not applicable because more than 50% of the tumor lesions were still stable until the time of the last follow-up. There was a significant difference in PFS between the TACE group and the TACE + MWA group (*P* = 0.003, Fig. 2A).

### OS

At the time of analysis, 15 patients (33.3%) had died and 30 (66.7%) were still alive. The 1-, 3-, and 5-year OS rates for the cohort were 93.2%, 64.5%, and 55.0%, respectively. In the TACE group, 12 patients (42.8%) had died at the time of analysis, and 1-, 3-, and 5-year OS rates were 89.0%, 58.1%, and 50.8%, respectively. In the TACE + MWA group, three patients (17.6%) had died at the time of analysis, and 1-, 3-, and 5-year OS rates were 100.0%, 82.1%, and 61.5%, respectively. There existed no statistical difference in OS rates between the groups (Fig. 2B).

### Safety evaluation

Based on the Score Index for Radiosurgery (SIR) grading system, several adverse events were noted and graded. Post-procedure minor complications (vomiting, fever, nausea and abdominal pain) occurred in 23.5% (4/17) of the patients in the TACE–MWA group and in 42.8% (12/28) in the TACE group. During the MWA procedure, most patients complained of local burning pain, but it was generally tolerable after use of basal anesthesia. After the procedure, some patients reported having fever and local pain, but these were not severe. There was no significant difference in the incidence of complications between the two groups (*P* = 0.219). All of these adverse effects were transient and were relieved after symptomatic treatment. No major complications (organ injury, hemorrhage, ascites, and liver failure) and death related to treatment were observed for either of the procedures.

## Discussion

In our retrospective analysis of 45 patients with recurrent small HCC, the 5-year PFS of patients receiving the combination therapy (37.5%) was higher than that of patients receiving TACE alone (18.7%, *P* = 0.003), indicating that the combination therapy showed better tumor response and may prolong tumor PFS time.

The combination treatment of TACE and ablation did not show better survival outcomes than ablation monotherapy in primary small HCC (Chai et al. 2021) [17], while the results remain vague in recurrent small HCC after resection. Our study demonstrated that TACE–MWA compared with TACE alone could offer better tumor control for patients with recurrent small HCC. Similar to the results from the study by Chen et al. [18], our study showed that 82.7% tumors were completely ablated in the TACE–MWA group, higher than 42.8% tumors in the TACE alone group at 1 month after the initial treatment (*P* = 0.013). Moreover, ORR and DCR were also significantly higher for the TACE–MWA group than the TACE group. Significant difference was found in PFS between the TACE and the TACE–MWA groups (*P* = 0.003),

The reason why TACE–MWA achieved better tumor control than TACE alone may lie in the synergistic effect of these two treatments. First, TACE could reduce the heat-sink effect of ablation by obstructing the tumor-feeding artery, thus in turn enhancing the coagulation effect of MWA. Second, chemotherapy agents may cause a heat-sensitizing effect and thermal injury may sensitize tumors to the chemotherapeutic agents [15]. Third, lipiodol was deposited in the tumor and peri-tumor margin after TACE, which could help to accurately assess the tumor size and facilitated the subsequent ablation. In addition, TACE also could help to control these micro-lesions, reducing the recurrence of tumor.

The cumulative OS rates at 5 years were 61.1% for TACE–MWA and 50.3% for TACE alone in recurrent sHCC patients, which is similar to previously reported 5-year cumulative OS rates of TACE-RFA (46–60%) [5, 13]. Although we found no significant difference in OS between the TACE group and the TACE–MWA group, complete necrosis after TACE has been linked to good survival outcome in patients with recurrent HCC [19]. A tendency favoring TACE–MWA was also found in our study. The findings of this study suggest that TACE could be used as an alternative treatment option for patients with small recurrence who are ineligible for curative therapies. Thus, our study may facilitate the development of a treatment algorithm for recurrent HCC.

There were some limitations in our study. First, the principal limitation was its susceptibility to selection bias owing to its retrospective nature. In the TACE group, most of the procedures were performed early in this study period, while most in the TACE–MWA group were performed later in this study period. Therefore, the influence of measured and unmeasured confounders on the outcome of patients was inevitable. Second, given the relatively small number of patients enrolled in this study, a larger number of subjects and a prospective study design are desirable to conform the results.

## Conclusion

In conclusion, TACE and TACE–MWA may lead to a similar OS in patients with recurrent small HCC after resection. However, TACE–MWA had better 1-, 3-, 6-month tumor response rates and may prolong tumor PFS time.

## Data Availability

The datasets generated and/or analysed during the current study are not publicly available, because it is related to subsequent research, but are available from the corresponding author upon reasonable request.

## Abbreviations

TACE: Transcatheter arterial chemoembolization
MWA: Microwave ablation
HCC: Hepatocellular carcinoma
PFS: Progression-free survival
OS: Overall survival
CR: Complete response
ORR: Objective response rate
DCR: Disease control rate
MRI: Magnetic resonance imaging
SD: Standard deviation;
IQR: Interquartile range
